# A98 FECAL LEUKOCYTE ESTERASE: AN ALTERNATIVE BIOMARKER TO FECAL CALPROTECTIN IN INFLAMMATORY BOWEL DISEASE

**DOI:** 10.1093/jcag/gwab049.097

**Published:** 2022-02-21

**Authors:** N K Klemm, R Trasolini, K Zhu, S Wong, B Salh

**Affiliations:** 1 University of British Columbia, Vancouver, BC, Canada; 2 Beth Israel Deaconess Medical Center, Boston, MA

## Abstract

**Background:**

Fecal calprotectin (FC) is a non-invasive biomarker used in inflammatory bowel disease (IBD) management and risk stratification of non-specific gastrointestinal symptoms. Leukocyte esterase is an inexpensive and widely available point-of-care inflammatory marker present on urinalysis test strips.

**Aims:**

We aim to assess the diagnostic accuracy of fecal leukocyte esterase (FLE) relative to FC and endoscopy and demonstrate its use as an alternative biomarker for IBD.

**Methods:**

In this prospective cohort study, 70 patients who had FC ordered as part of standard clinical care also received FLE testing. FLE levels were compared to various FC cut-off values, endoscopy and pathology findings as gold standard.

**Results:**

As the FC cut-off increased from 50 to 500 μg/g, FLE sensitivity increased from 67% to 95% while the specificity decreased from 86% to 76%. The area under the receiver operating characteristic (AUROC) increased from 0.79 to 0.90. An FLE of ≥1+ had the best test characteristics. Amongst patients who underwent endoscopic evaluation, FLE demonstrated an identical sensitivity (75%) and specificity (86%) to FC in predicting endoscopic inflammation. AUROC was 0.80 for FLE and 0.85 for FC with an optimal cut-off of ≥2+ and 301 μg/g, respectively. When used to distinguish between active IBD and no/inactive IBD patients, FLE had a sensitivity of 84% and specificity of 90%, comparable to the 84% and 83%, respectively, of FC. AUROC was 0.88 for FLE and 0.91 for FC with an optimal cut-off of ≥2+ and 145 μg/g, respectively

**Conclusions:**

FLE demonstrates adequate correlation and comparable accuracy to FC in predicting endoscopic inflammation and distinguishing between patients with active versus inactive IBD.

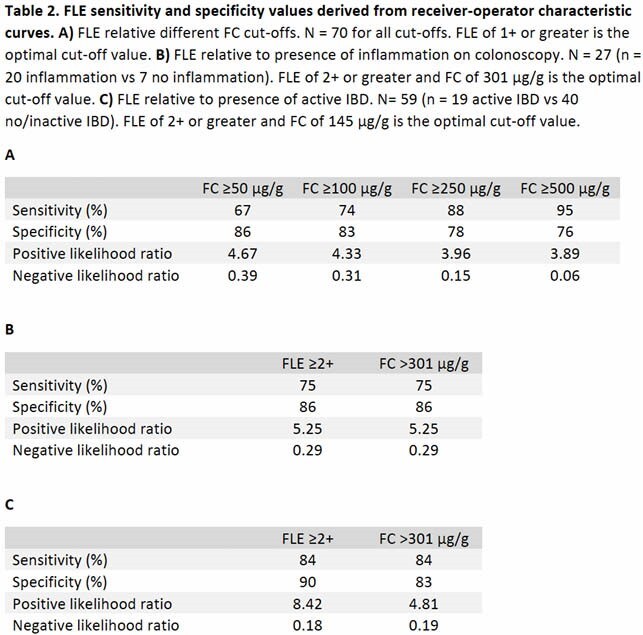

**Funding Agencies:**

None

